# Dynamic fluorescent imaging with the activatable probe, γ-glutamyl hydroxymethyl rhodamine green in the detection of peritoneal cancer metastases: Overcoming the problem of dilution when using a sprayable optical probe

**DOI:** 10.18632/oncotarget.9898

**Published:** 2016-06-07

**Authors:** Yuko Nakamura, Toshiko Harada, Tadanobu Nagaya, Kazuhide Sato, Shuhei Okuyama, Peter L. Choyke, Hisataka Kobayashi

**Affiliations:** ^1^ Molecular Imaging Program, Center for Cancer Research, National Cancer Institute, Bethesda, MD 20892, USA

**Keywords:** kinetic map, green emitting probe, autofluorescence, γ-glutamyltranspeptidase, peritoneal cancer metastases

## Abstract

Optical fluorescence-guided imaging is increasingly used to guide surgery and endoscopic procedures. Activatable probes are particularly useful because of high target-to-background ratios that increase sensitivity for tiny cancer foci. However, green fluorescent activatable probes suffer from interference from autofluorescence found in biological tissue. The purpose of this study was to determine if dynamic imaging can be used to differentiate specific fluorescence arising from an activated probe in a tumor from autofluorescence in background tissues especially when low concentrations of the dye are applied. Serial fluorescence imaging was performed using various concentrations of γ-glutamyl hydroxymethyl rhodamine green (gGlu-HMRG) which was sprayed on the peritoneal surface with tiny implants of SHIN3-DsRed ovarian cancer tumors. Temporal differences in signal between specific green fluorescence in cancer foci and non-specific autofluorescence in background tissue were measured at 5, 10, 20 and 30 min after application of gGlu-HMRG and were processed into three kinetic maps reflecting maximum fluorescence signal (MF), wash-in rate (WIR), and area under the curve (AUC), respectively. Using concentrations up to 10 μM of gGlu-HMRG, the fluorescence intensity of cancer foci was significantly higher than that of small intestine but only at 30 min. However, on kinetic maps derived from dynamic fluorescence imaging, the signal of cancer foci was significantly higher than that of small intestine after only 5 min even at concentrations as low as 2.5 μM of gGlu-HMRG (*p* < 0.01). At lower concentrations, kinetic maps derived from dynamic fluorescence imaging were superior to unprocessed images for cancer detection.

## INTRODUCTION

In many oncologic procedures, the ability to completely resect tumors is important for long term durable responses. Although large tumors are visible to the unaided human eye and can be readily removed, tiny foci (2 to 3 mm) of cancer metastases or invading cells may be more difficult to see. Consequently, optical fluorescence-guided imaging is increasingly used as an aid to surgery or endoscopy to guide the detection of tiny tumor foci. Optical fluorescence imaging offers high sensitivity, low cost, portability, real-time capabilities, and importantly, absence of ionizing radiation [1–5] and thus, there is much interest in this topic.

There are two major categories of fluorescent probes that have been used in this context: ‘always on’ and activatable probes [6]. Always-on probes fluoresce regardless of whether they are bound to the target tissue and thus, have the disadvantage of high background signal. One approach is to wait for clearance of background signal so that adequate target-to-background ratios (TBRs) can be achieved but signal within the tumor also decreases, lowering sensitivity. On the other hand, activatable probes only become fluorescent after they come in contact with the target tissue. Thus, this class of optical probes have lower background signals, but require rapid activation to be practical in the clinical environment [7]. One common approach for activating optical probes is to make use of specific enzymatic activity found in the tumor microenvironment but not in normal tissues [8].

γ-glutamyl hydroxymethyl rhodamine green (gGlu-HMRG) is an activatable optical probe that produces the green fluorescent product, HMRG, after exposure to γ-glutamyltranspeptidase (GGT), a cell surface-associated (or bound) enzyme involved in cellular glutathione homeostasis. GGT is overexpressed in several human tumors, including cervical and ovarian cancers [9–13]. gGlu-HMRG has been reported to be able to detect intraperitoneal metastases in preclinical mouse models within 10 min of topical application because of its rapid and strong activation upon contact with GGT [9]. This probe is also sprayable onto the surface of tumors, making it quite convenient to deliver to an entire surgical or endoscopic field. Several studies have assessed the diagnostic performance of gGlu-HMRG for various cancers and more studies are underway [14–17].

The choice of emission wavelength for an optical probe depends on use for which it is intended. For lesions that might be hiding beneath the organ surface, near infrared light is preferred because of its depth of penetration in tissue. However, for surgical or endoscopic procedures, where the task is to detect surface lesions, shorter wavelengths can be considered. Green light is of interest because the human eye is exquisitely sensitive to it and it requires no special equipment for detection. However, green light also poses some challenges, particularly, interference from autofluorescence. In the case of detecting intraperitoneal metastases, autofluorescence of surrounding normal tissue, such as the small bowel, may hamper the detection of intraperitoneal lesions. This is especially true if the probe becomes diluted in pools of fluid in the body cavity and thus, emits lower intensity of light. One approach to reducing autofluorescence is to unmix known autofluorescence spectra from the optical probe spectra. However, current spectral imaging is time consuming requiring at least several seconds per frame and therefore, is not amenable to real time imaging during surgical or endoscopic procedures [6, 18].

One difference between the green light from an exogenous activatable fluorophore and endogenous autofluorescence is that the former is dynamic in signal characteristics while the latter is generally constant. Thus, evaluation of dynamic changes of fluorescence signal after application of an activatable probe is a potential method to differentiate between fluorescence from HMRG on cancer foci and autofluorescence in surrounding normal tissue over a wide range of dye concentrations.

**Figure 1:**
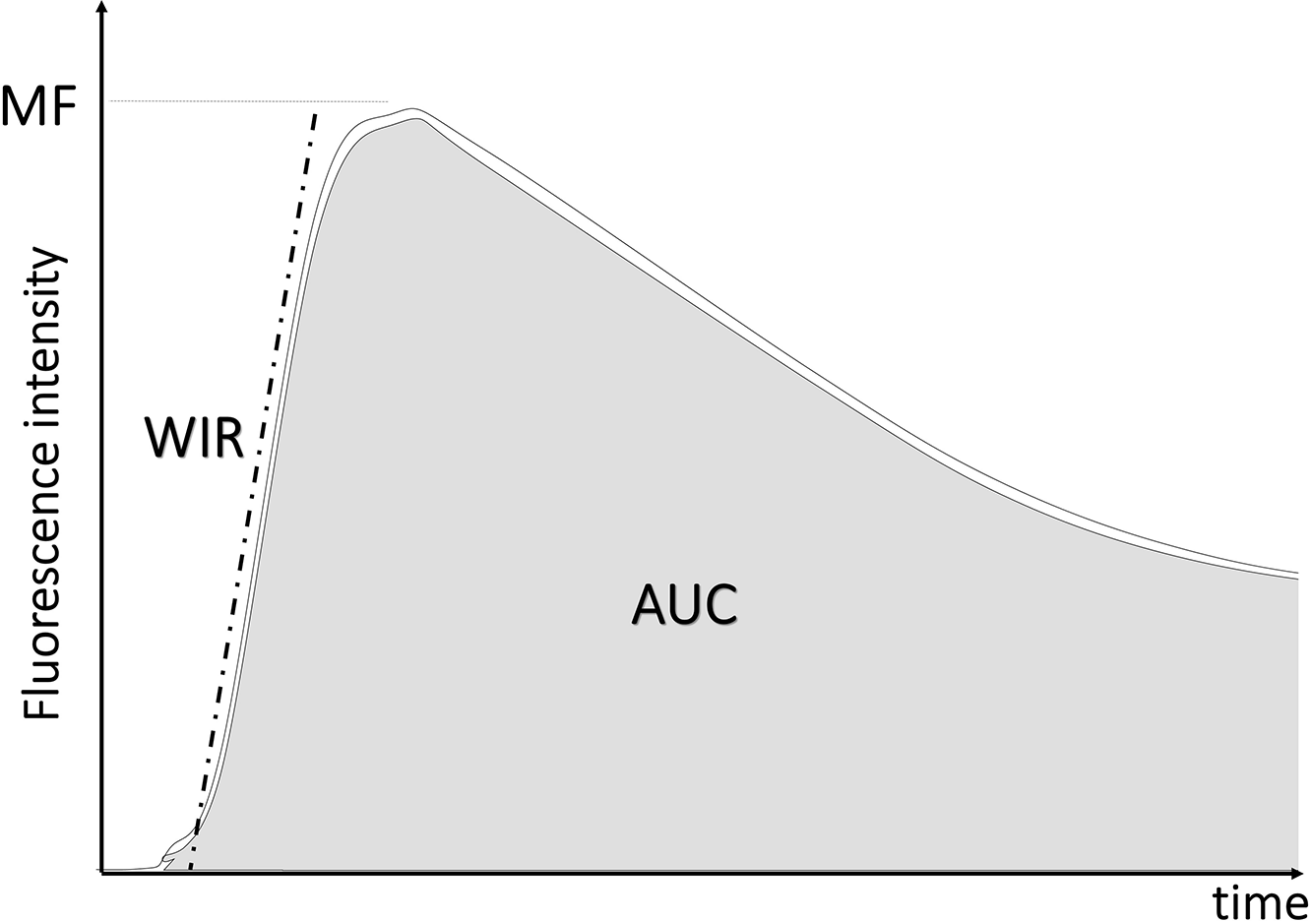
Schema of creating kinetic maps

In this study, we used the sprayable activatable probe, gGlu-HMRG in an animal model of peritoneal ovarian cancer metastases (POCM) and acquired dynamic fluorescence images at varying concentrations of the dye. Using dynamic images we created kinetic maps based on calculated three parameters (Figure 1), maximum fluorescence signal (MF), wash-in rate (WIR), and area under the curve (AUC), which are frequently used as semi-quantitative parameters in conventional dynamic imaging of contrast-enhanced MRI and nuclear medicine especially for differentiating between benign and malignant tumors [19–22]. MF is the maximum fluorescence signal observed during the entire dynamic images. WIR is the maximum slope approaching the MF. AUC is the area measured under the time-fluorescence curve. We investigated the utility of kinetic maps to differentiate between cancer foci and background tissue.

## RESULTS

To simulate the effects of dilution, a variety of concentrations of gGlu-HMRG were utilized resulting in the following observations:

### 2.5 uM gGlu-HMRG

**Figure 2:**
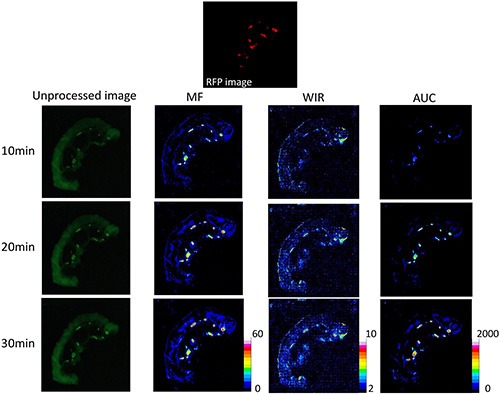
Unprocessed images and kinetic maps (MF, WIR, and AUC maps) using 2.5 μM gGlu-HMRG, and RFP image (the standard of reference for cancer location) The detection of cancer foci was more straightforward on MF and AUC maps compared to unprocessed images.

Fluorescence intensity of cancer foci increased gradually over 30 min after spraying gGlu-HMRG on the specimen (*p* = 0.09 at 5 min and < 0.01 at 10, 20, and 30 min after gGlu-HMRG, respectively). On the other hand, fluorescence intensity of the small intestine did not change after spraying gGlu-HMRG and there was no significant difference (*p* = 0.41, 0.47, 0.41 and 0.30 at 5, 10, 20 and 30 min after spraying gGlu-HMRG, respectively) (Figure 2, Supplementary Figures S1, S2 and Supplementary Video S1).

On unprocessed images, the fluorescence intensity of cancer foci was significantly higher than that of small intestine only at 30 min after spraying gGlu-HMRG (*p* = 0.42, 0.63, 0.08 and 0.04 at 5, 10, 20 and 30 min after spraying gGlu-HMRG, respectively). On the other hand, maximum fluorescence signal (MF), wash-in rate (WIR), and area under the curve (AUC) were significantly higher in cancer foci than small intestine at all time points even as early as 5 minutes (*p* < 0.01 at all time point for all three parameters) (Figures 2, 7, and Supplementary Figures S3–S6).

### 5 uM gGlu-HMRG

**Figure 3:**
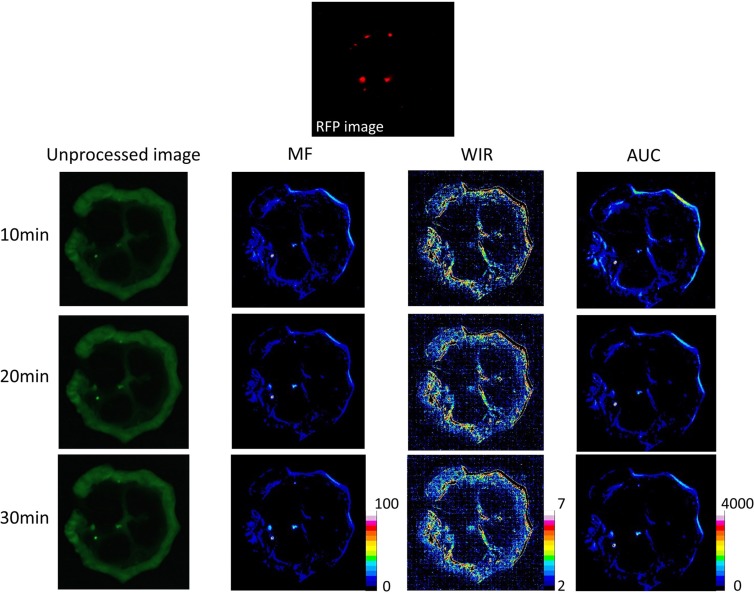
Unprocessed images and kinetic maps (MF, WIR, and AUC maps) using 5 μM gGlu-HMRG, and RFP image (the standard of reference for cancer location) The detection of cancer foci was a little easier on MF and AUC maps compared to unprocessed images.

Fluorescence intensity of cancer foci increased gradually up to 30 min after spraying gGlu-HMRG (*p* = 0.30 and 0.04 at 5 and 10 min, < 0.01 at 20 and 30 min after spraying gGlu-HMRG, respectively). On the other hand, the fluorescence intensity of small intestine did not change after spraying gGlu-HMRG and there were no significant differences (*p* = 0.76, 0.75, 0.69 and 0.60 at 5, 10, 20 and 30 min after spraying gGlu-HMRG, respectively) (Figure 3, Supplementary Figures S1, S2 and Supplementary Video S2).

On unprocessed images the fluorescence intensity of cancer foci was significantly higher than that of small intestine only at 30 min after spraying gGlu-HMRG (*p* = 0.20, 0.75, 0.15 and 0.02 at 5, 10, 20 and 30 min after spraying gGlu-HMRG, respectively). On the other hand, MF of cancer foci was significantly higher than that of small intestine at all time points (*p* = 0.02 at 5 min and *p* < 0.01 at 10, 20, and 30 min, respectively). AUC of cancer foci was significantly higher than that of small intestine at time points 10 min and greater (*p* = 0.15 at 5 min and *p* < 0.01 at 10, 20, and 30 min after spraying gGlu-HMRG, respectively). WIR of cancer foci tended to be higher compared to that of small intestine. However, there was no significant difference (*p* = 0.52, 0.75, 0.26 and 0.07 at 5, 10, 20 and 30 min after spraying gGlu-HMRG, respectively) (Figures 3, 8 and Supplementary Figures S3–S6).

### 10 uM gGlu-HMRG

**Figure 4:**
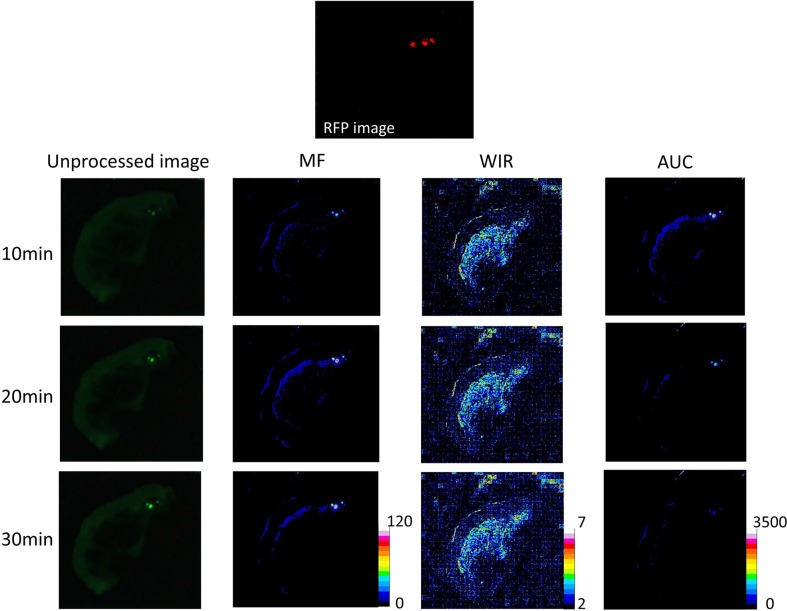
Unprocessed images and kinetic maps (MF, WIR, and AUC maps) using 10 μM gGlu-HMRG, and RFP image (the standard of reference for cancer location) The detection of cancer foci on unprocessed images and kinetic maps was almost identical visually.

Fluorescence intensity of cancer foci increased gradually up to 30 min after spraying gGlu-HMRG (*p* = 0.47 and 0.05 at 5 and 10 min, < 0.01 at 20 and 30 min after spraying probe, respectively). On the other hand, fluorescence intensity of the small intestine did not change after spraying gGlu-HMRG and there were no significant differences (*p* = 0.79, 0.81, 0.77 and 0.77 at 5, 10, 20 and 30 min after spraying gGlu-HMRG, respectively) (Figure 4, Supplementary Figures S1, S2 and Supplementary Video S3).

On unprocessed images fluorescence intensity of cancer foci was significantly higher than that of small intestine only at 30 min after spraying gGlu-HMRG (*p* = 0.63, 0.42, 0.11 and 0.01 at 5, 10, 20 and 30 min after spraying gGlu-HMRG, respectively). On the other hand, MF and AUC of cancer foci was significantly higher than that of small intestine at all time points (*p* = 0.02 at 5 min and *p* < 0.01 at 10, 20, and 30 min after spraying gGlu-HMRG for MF, *p* = 0.02 at 5 min and *p* < 0.01 at 10, 20, and 30 min after spraying gGlu-HMRG for AUC, respectively). WIR of cancer foci tended to be higher compared to that of small intestine with a significant difference at 20 and 30 min after spraying gGlu-HMRG (*p* = 0.20, 0.08, 0.04 and 0.02 at 5, 10, 20 and 30 min after spraying gGlu-HMRG, respectively) (Figures 4, 9 and Supplementary Figures S3–S6).

### 20 uM gGlu-HMRG

**Figure 5:**
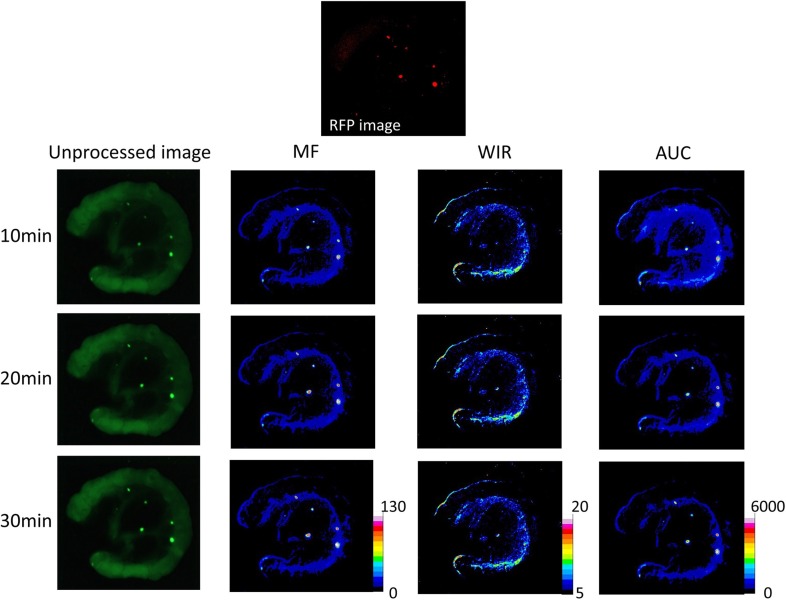
Unprocessed images and kinetic maps (MF, WIR, and AUC maps) using 20 μM gGlu-HMRG, and RFP image (the standard of reference for cancer location) The detection of cancer foci on unprocessed images and kinetic maps was almost identical visually. However, autofluorescence of small intestine was prominent on unprocessed images.

Fluorescence intensity of cancer foci increased gradually up to 30 min after spraying gGlu-HMRG (*p* = 0.22 and 0.01 at 5 and 10 min, < 0.01 at 20 and 30 min after spraying gGlu-HMRG, respectively). On the other hand, fluorescence intensity of the small intestine did not change after spraying gGlu-HMRG and there was no significant difference (*p* = 0.69, 0.71, 0.70 and 0.69 at 5, 10, 20 and 30 min after spraying gGlu-HMRG, respectively) (Figure 5, Supplementary Figures S1, S2 and Supplementary Video S4).

On unprocessed images fluorescence intensity of cancer foci tended to be higher than that of small intestine with significant differences at 20 and 30 min after spraying gGlu-HMRG (*p* = 0.52 and 0.33 at 5 and 10 min, < 0.01 at 20 and 30 min after spraying gGlu-HMRG, respectively). All three parameters, MF, WIR and AUC of cancer foci were significantly higher than that of small intestine at all time points (*p* < 0.01 at all time points for all three parameters) (Figures 5, 10 and Supplementary Figures S3–S6).

### 100 uM gGlu-HMRG

**Figure 6:**
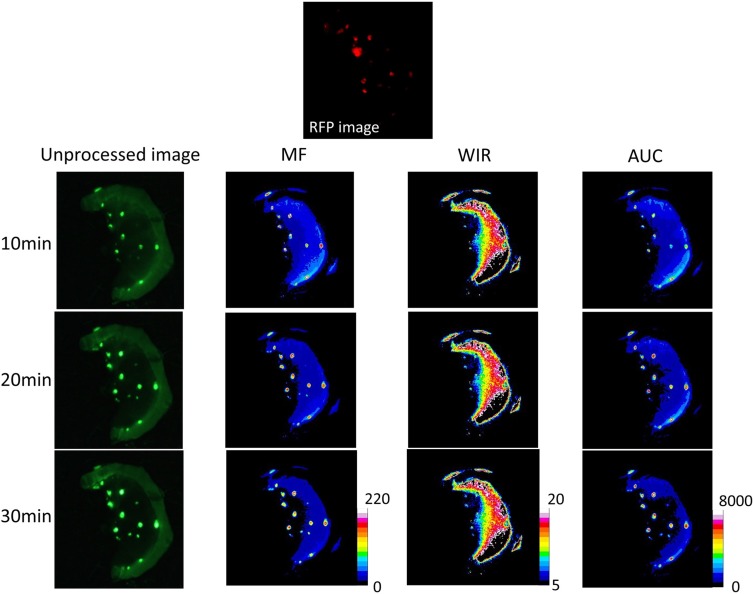
Unprocessed images and kinetic maps (MF, WIR, and AUC maps) using 100 μM gGlu-HMRG, and RFP image (the standard of reference for cancer location) Cancer foci were clearly detected on the unprocessed images. On the other hand, small bowel mesentery showed high fluorescence signal on WIR maps potentially obscuring some lesions.

Spraying gGlu-HMRG resulted in a marked increase of fluorescence intensity of cancer foci followed by gradual increase up to 30 min (*p* < 0.01 at all time points). On the other hand, fluorescence intensity of small intestine did not change after spraying gGlu-HMRG and there were no significant differences (*p* = 0.41, 0.47, 0.41 and 0.30 at 5, 10, 20 and 30 min after spraying gGlu-HMRG, respectively) (Figure 6, Supplementary Figures S1, S2 and Supplementary Video S5).

On unprocessed images fluorescence intensity of cancer foci was higher than that of small intestine with significant differences at all time points (*p* < 0.01 at all time points). All three parameters, MF, WIR and AUC of cancer foci were also significantly higher than that of small intestine at all time points (*p* < 0.01 at all time points for all three parameters) (Figures 6, 11 and Supplementary Figures S3–S6). However, high signal on small bowel mesentery hampered the evaluation of cancer foci on the WIR color map.

**Figure 7:**
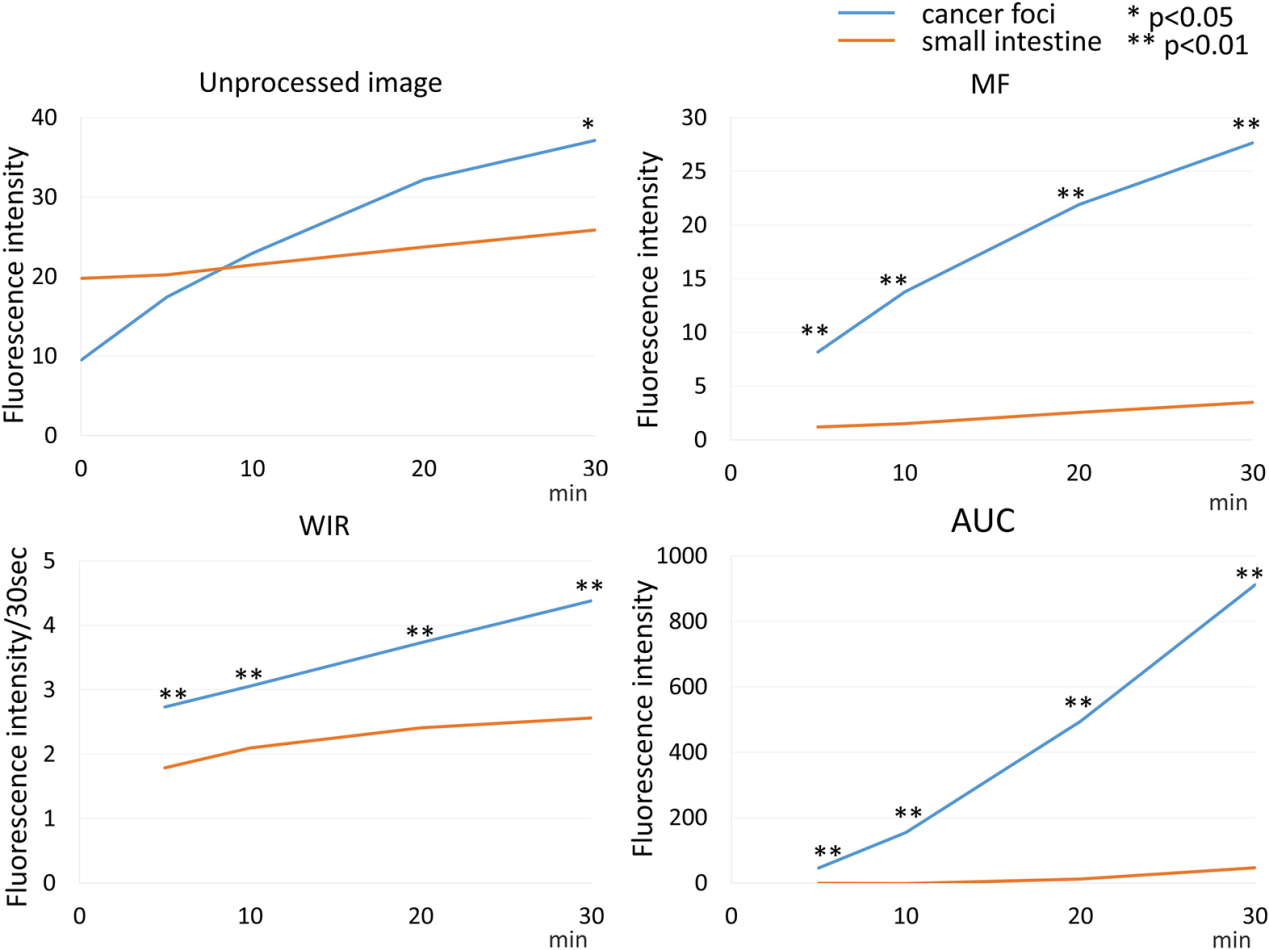
Time fluorescence intensity curve of the cancer foci and small intestine on unprocessed images, MF, WIR, and AUC maps using 2.5 μM gGlu-HMRG Difference between cancer foci and small intestine was examined at each time point.

**Figure 8:**
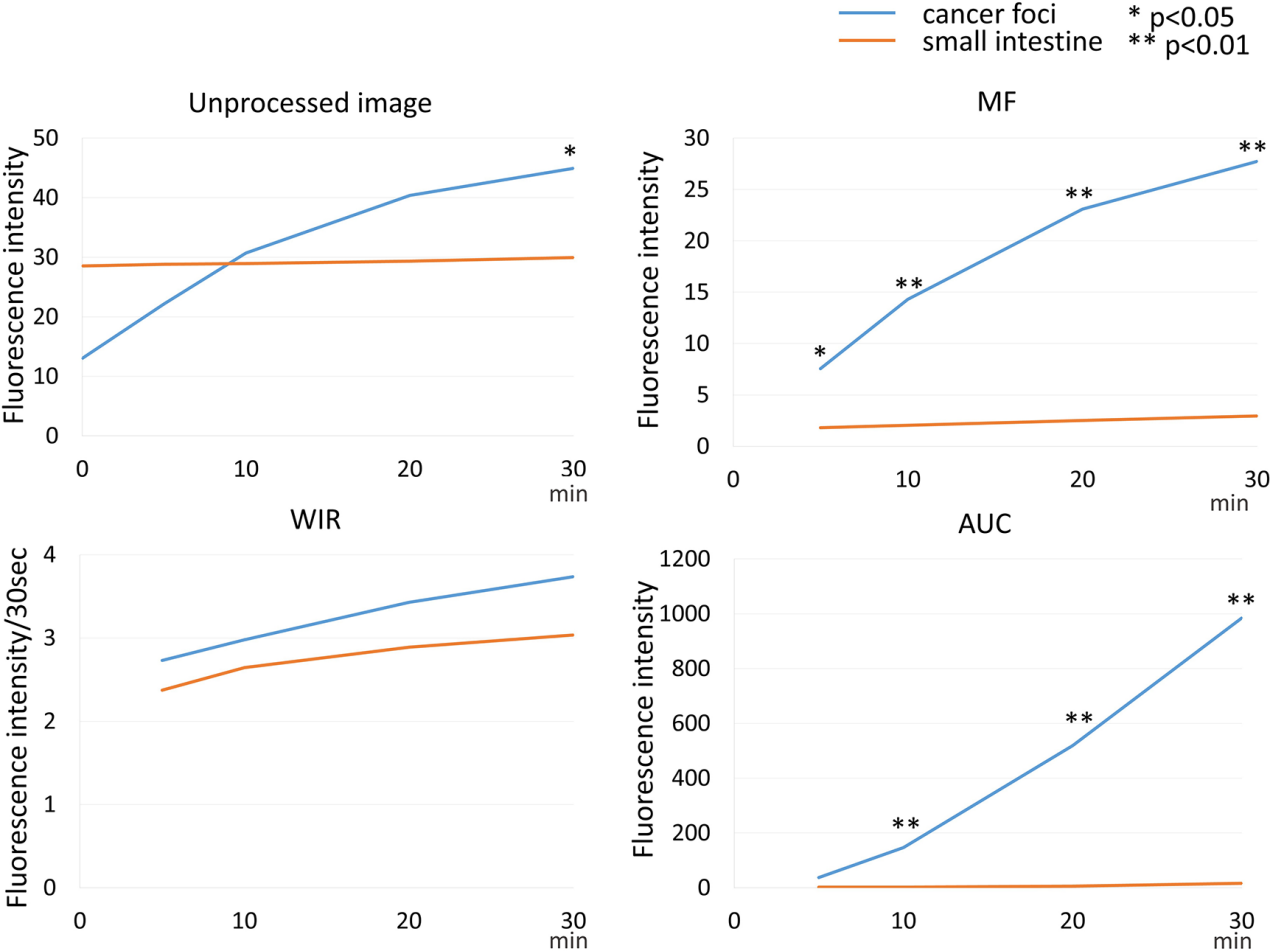
Time fluorescence intensity curve of the cancer foci and small intestine on unprocessed images, MF, WIR, and AUC maps using 5 μM gGlu-HMRG Difference between cancer foci and small intestine was examined at each time point.

**Figure 9:**
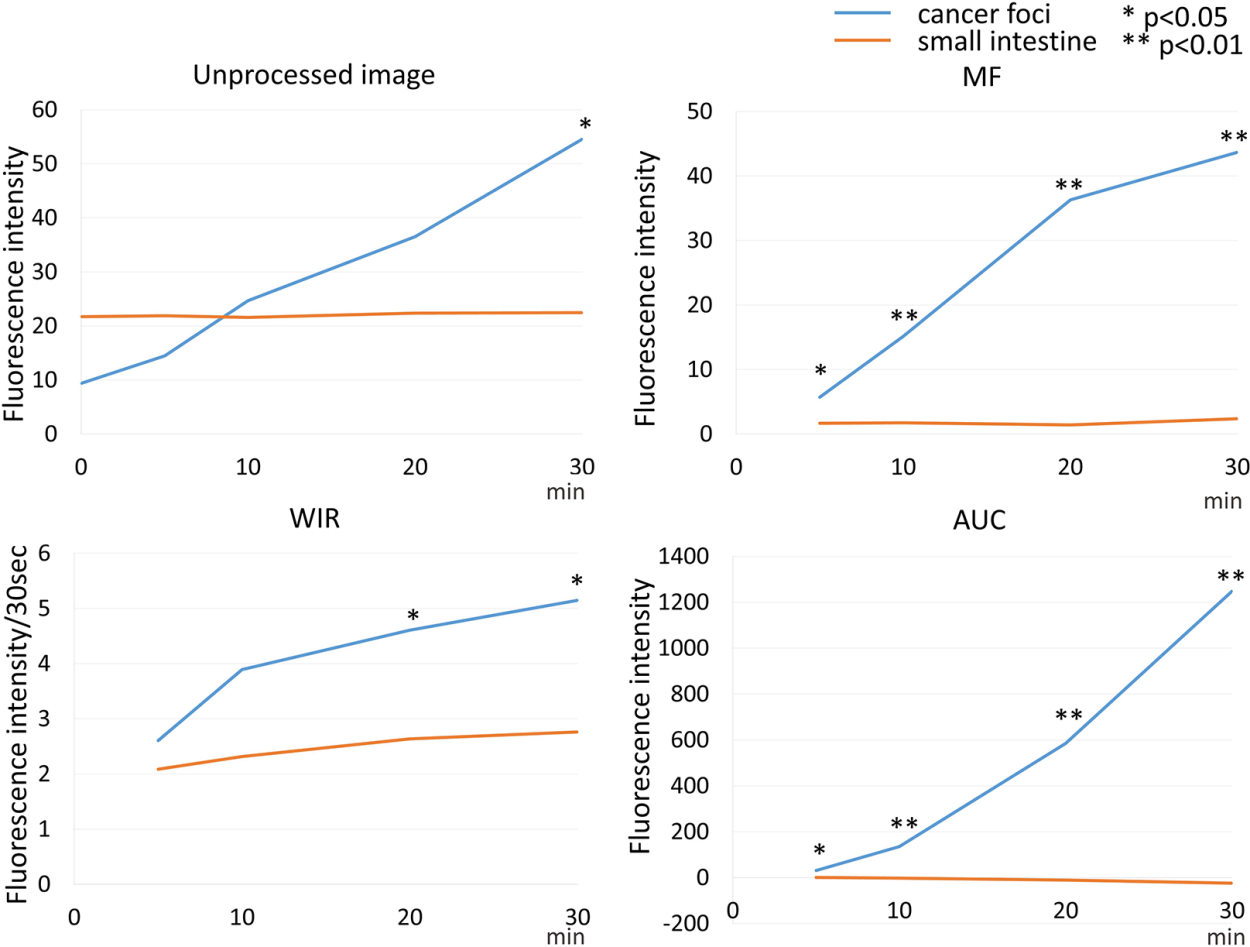
Time fluorescence intensity curve of the cancer foci and small intestine on unprocessed images, MF, WIR, and AUC maps using 10 μM gGlu-HMRG Difference between cancer foci and small intestine was examined at each time point.

**Figure 10:**
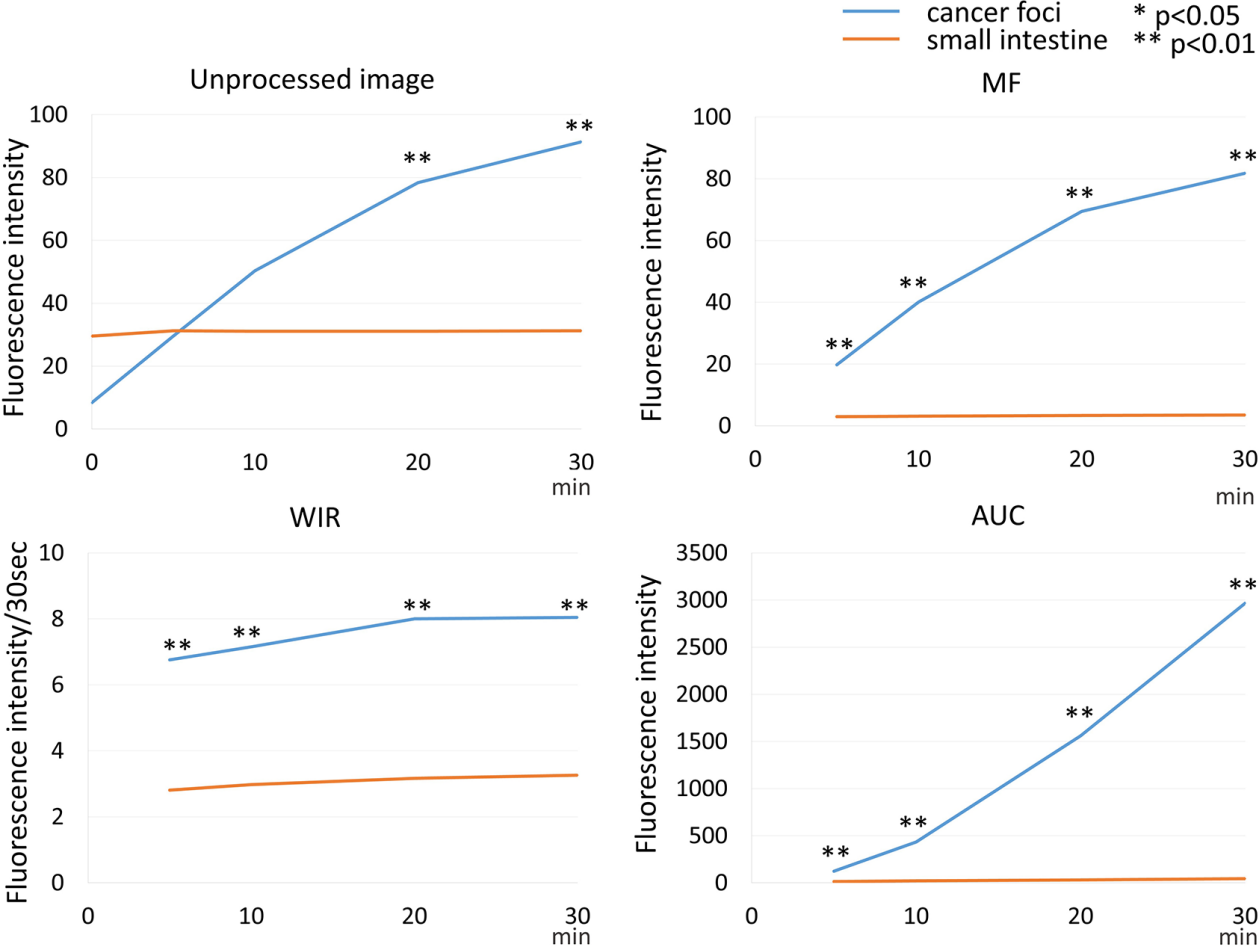
Time fluorescence intensity curve of the cancer foci and small intestine on unprocessed images, MF, WIR, and AUC maps using 20 μM gGlu-HMRG Difference between cancer foci and small intestine was examined at each time point.

**Figure 11:**
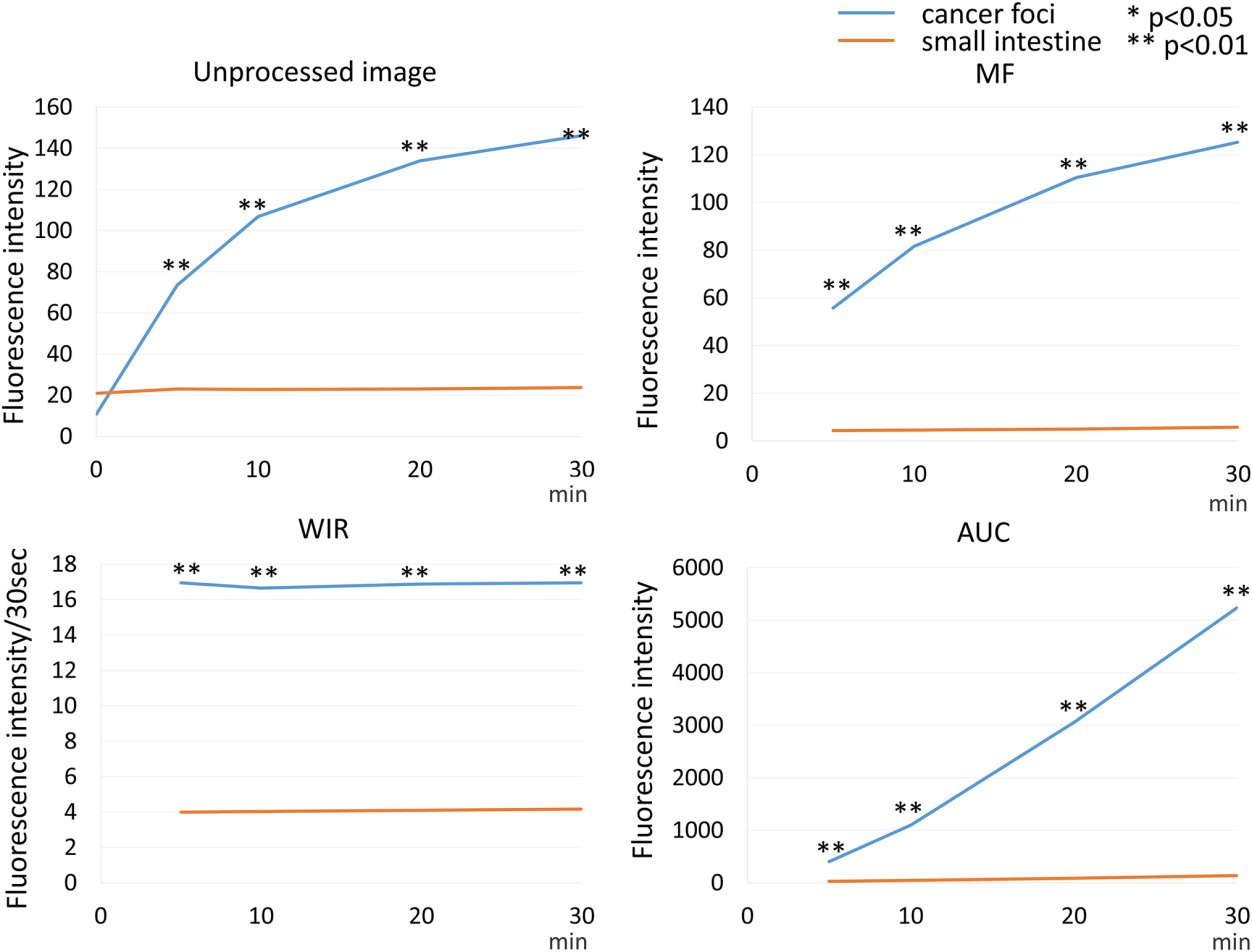
Time fluorescence intensity curve of the cancer foci and small intestine on unprocessed images, MF, WIR, and AUC maps using 100 μM gGlu-HMRG Difference between cancer foci and small intestine was examined at each time point.

### Comparison of tumor: small intestine ratio at an early time point

**Figure 12:**
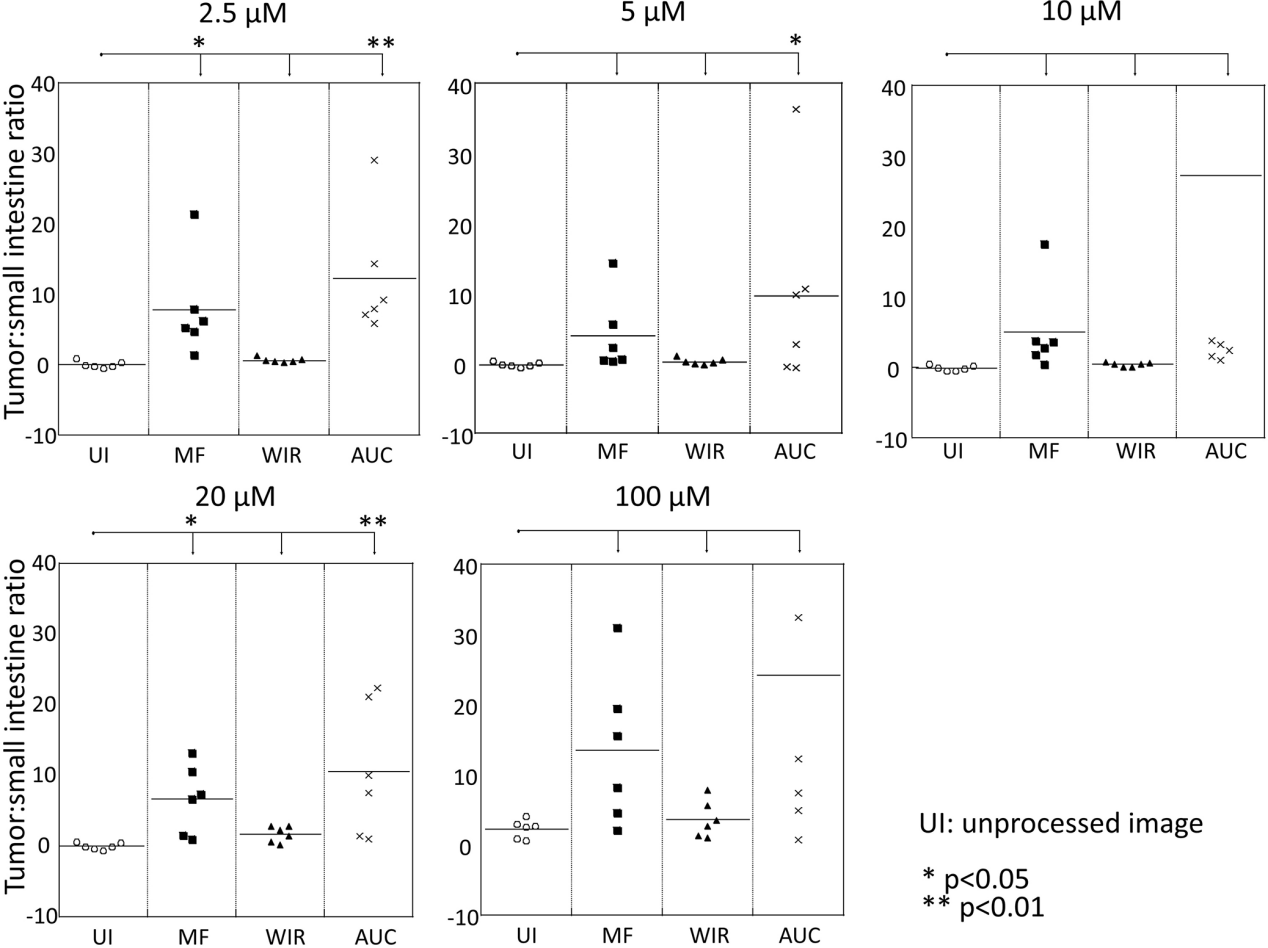
Comparison of tumor:small intestine ratio of three parameters, MF, WIR, and AUC, compared to that of unprocessed image

Tumor:small intestine ratio (T:SI) of unprocessed images was approximately zero up to 20 μM. On the other hand, T:SI of the three parameters tended to demonstrate higher ratios compared to ratios of unprocessed images regardless of concentration of gGlu-HMRG (Figure 12). Using 2.5 μM and 20 μM gGlu-HMRG, the T:SI using MF and AUC was significantly higher than the T:SI using unprocessed images (*p* = 0.03, 0.68 and < 0.01 for MF, WIR, and AUC at 2.5 μM, and *p* = 0.05, 0.52 and < 0.01 for MF, WIR, and AUC at 20 μM, respectively). Using 5 μM gGlu-HMRG the T:SI of the AUC was significantly higher than that of unprocessed images (*p* = 0.35, 0.71 and 0.04 for MF, WIR, and AUC, respectively). Using 10 μM and 100 μM gGlu-HMRG there was no significant difference in T:SI for all three parameters compared to that of unprocessed images (*p* = 0.63, 0.74 and 0.15 for MF, WIR, and AUC at 10 μM, and *p* = 0.29, 0.70 and 0.05 for MF, WIR, and AUC at 100 μM, respectively).

## DISCUSSION

At all concentrations, fluorescence intensity of HMRG in cancer foci increased continually over 30 min after spraying gGlu-HMRG on the surface. Meanwhile, fluorescence intensity of the small intestine did not change. These results indicate that gGlu-HMRG was specifically activated by cancer even at low concentrations and was specifically activated by the tumor microenvironment even at high concentrations.

On unprocessed images, the fluorescence intensity of cancer foci was significantly higher than that of small intestine only at 30 min after spraying gGlu-HMRG when the concentration of the dye was up to 10 μM. This is likely due to the fact that at lower concentrations of the dye fluorescence was comparable in intensity to autofluorescence but above 10 μM the dye produced stronger fluorescence compared to autofluorescence.

However, kinetic maps were able to discern the presence of HMRG within cancer foci even at lower concentrations of the dye. For instance, the MF map showed that cancers were significantly higher in signal than small intestine at all time points even using 2.5 μM, the lowest concentration of gGlu-HMRG in the study. The AUC map also showed that cancers were significantly higher than small intestine at all time points. Thus, the dynamic parameters MF and AUC, were useful in overcoming the effects of autofluorescence and may be especially useful with low concentrations of gGlu-HMRG as might occur due to uneven dilution in fluids pooling in a body cavity.

The WIR map tended to demonstrate higher signal in cancers compared to small intestine regardless of concentration of gGlu-HMRG. However, the WIR produced inconsistent results at several time points indicating it is a less robust parameter than MF and AUC. For instance, there was no significant difference in WIR between cancer foci and small intestine at all time points with 5 μM gGlu-HMRG. WIR is reflective of the maximum slope of fluorescence intensity curve on subtracted images and may reflect presence of GGT within the tumor [9]. The exact function of GGT and how its presence benefits the tumor is still unclear [23]. However, GGT has also been reported to be overexpressed in several human tumors, including those from cervical and ovarian cancers [9–12] and is thought to promote tumor progression, invasion, and drug resistance, possibly through modulation of the intracellular redox metabolism [24]. Thus, WIR has the potential to be an imaging biomarker for GGT expression but may be less useful as a means of detecting low levels of HMRG.

When the highest concentration, 100 μM gGlu-HMRG, was used, cancer foci could be discerned at all time points on unprocessed images. The MF and AUC maps were also positive but were superfluous to the unprocessed images. The WIR map had high overall signal hampering the detection of cancer foci thus, confirming it is the least useful kinetic parameter. The gGlu-HMRG solution with high concentration could be activated by a minimal amount of GGT extruded from cancer foci to adjacent mesentery and emit fluorescence signal on mesentery shortly after spraying gGlu-HMRG. Attention must be paid to this issue when using the WIR map with high concentration gGlu-HMRG.

Thus, these data suggested that kinetic maps, especially those based on MF and AUC, permitted detection of cancer foci across a wide range of dye concentrations including low concentrations where unprocessed images were unhelpful at early time points. At high concentrations of gGlu-HMRG cancer foci could be clearly discerned on the unprocessed images. Thus, unprocessed images are most useful at high concentrations and kinetic maps are most useful at low concentrations and their combined use is therefore, complementary given that the dye is likely to get diluted in an unpredictable manner due to pools of fluid in body cavities (e.g. ascites, irrigation fluid etc.). These findings are of importance in improving the performance of dyes used in image guided surgical or endoscopic procedures. Most probes, including gGlu-HMRG, have been developed to aid surgeons in detecting tiny cancer foci, delineating the borders of tumors for complete removal and confirming the absence of residual tumor [9, 14–16]. The probe may be distributed on the surface of the target tissue in an inhomogeneous manner and the concentration of probe may vary with location. Thus, the combination of unprocessed images and kinetic maps, which detect cancer foci regardless of dye concentration, is considered to be a superior method for detection of cancer foci clinically.

Classically, fluorescence from a specific probe has been detected with multispectral imaging, which is the most sensitive optical technique for the identification of target tumors. However, current multispectral imaging takes at least 10 seconds per frame using expensive specially-resolved filter devices for scanning sufficient range of spectra and therefore, is not amenable to surgical or endoscopic procedures [6, 18]. Kinetic parametric maps, such as MF or AUC, offer superior contrast between tumor and small intestine before target tumors show up with sufficient fluorescence intensity on unprocessed images. Thus, this simple image processing of dynamic fluorescence imaging can assist tumor detection on unprocessed images obtained by inexpensive regular video camera. Moreover, kinetic maps can be created almost real-time with an appropriate image processing program because of the simple processing, suggesting that they could be useful for detection of hard-to-see tumors in intraoperative setting.

A reported approach for reducing autofluorescence is to use narrow bandwidth filters [25–29]. In this camera system, we employed a similar narrow bandwidth filter setting that is appropriate for excitation and emission of HMRG. However, when applied low concentration of gGlu-HMRG, signal from tumors was difficult to be detected especially at short time after administration because the signal from gGlu-HMRG was quickly increasing but still lower than that from fluorescent proteins including green fluorescent protein (GFP) which emits strong fluorescence signal [30–39]. Kinetic maps helped detecting such hard-to-see HMRG signal yielded from tumors with regular video cameras. Thus, these kinetic maps are useful especially for detecting low fluorescence signal derived from probe submerged in autofluorescence of surrounding normal tissue.

One important limitation in kinetic maps is the mis-registration. Kinetic maps are created by processing serial images which are taken different time after spraying probes. Perfect subtraction is sometimes difficult because of the slight movement of fresh samples at each time point due to natural contraction or evaporation of fluid. Incomplete subtraction especially at the edge of fluorescent objects might cause a potential error on post-processed parameters because of a large change in fluorescence signal. Indeed, cancer foci adjacent to small intestine confirmed on RFP images was sometimes difficult to detect on kinetic maps when using low concentration of gGlu-HMRG because false high values on kinetic maps around small intestine due to mis-registration hampered the detection of cancer foci (see Figure 3). Attention must be paid to this issue when a kinetic map is read.

In conclusion, kinetic maps were useful in overcoming autofluorescence especially with low concentrations of gGlu-HMRG. While cancer foci were detected clearly on unprocessed images at high concentrations of gGlu-HMRG, kinetic maps were superior when low concentrations of the dye were present. Thus, the combination of unprocessed images and kinetic maps is potentially important for detecting cancer foci regardless of concentration of gGlu-HMRG during surgical or endoscopic procedures.

## MATERIALS AND METHODS

### Reagents

gGlu-HMRG, a rapidly activatable cancer-selective fluorescence imaging probe, was synthesized as described previously [9].

### Cell lines and culture

SHIN3, is an ovarian cancer cell line that highly expresses GGT and shows strongly positive fluorescent signal with gGlu-HMRG [9]. SHIN3-DsRed contains a red fluorescent protein (RFP DsRed2)-expressing plasmid (Clontech Laboratories) that was stably transfected into SHIN3 cells to enable detection on *ex vivo* optical imaging of POCM [40]. Cell lines were grown in RPMI 1640 supplemented with 10% FBS and 1% penicillin-streptomycin in tissue culture flasks in a humidified incubator at 37°C in an atmosphere of 95% air and 5% carbon dioxide.

### Animal model

All procedures were performed in compliance with the Guide for the Care and Use of Laboratory Animals [41] and approved by the local Animal Care and Use Committee. Six- to 8-week old female homozygote athymic nude mice were purchased from Charles River (National Cancer Institute, Frederick, MD).

To generate the animal model, intraperitoneal xenografts were established by intraperitoneal (i.p.) injection of 2 × 10^6^ SHIN3-DsRed cells suspended in 200 to 300 μl of phosphate-buffered saline (PBS) into the peritoneal cavity of nude mice. Imaging was performed at 14–21 days after injection of the cells.

### *Ex vivo* activatable imaging

gGlu-HMRG stock solution (containing 0.5% v/v DMSO as a co-solvent) was suspended in PBS to generate the following concentrations: 2.5, 5, 10, 20, and 100 μM gGlu-HMRG solution which were used to simulate dilution in a clinical situation. Mice with tumors were euthanized by carbon dioxide inhalation. Immediately after euthanasia, the mouse abdominal wall was incised, and the abdominal cavity was exposed. The small bowel, its mesentery and any POCM were extracted *en bloc.*

To examine the dynamic changes in green fluorescence intensity over time, serial fluorescence imaging was performed after gGlu-HMRG was sprayed on the specimen. A portable fluorescence camera (Discovery INDEC BioSystems, Santa Clara, CA, USA) was utilized [42] with the following filter set: band-pass filter from 450 to 490 nm for excitation light and from 511 to 551 nm for emission light, with an exposure time of 50 msec. Extracted specimens were placed on a non-fluorescent plate. After baseline images were obtained, a 100 μl solution of gGlu-HMRG (2.5, 5, 10, 20, and 100 μM, respectively) was sprayed on a dry specimen surface. Real-time fluorescence images were recorded every 30 sec between 0 and 30 min after gGlu-HMRG administration.

For evaluation of red fluorescence indicating the presence of tumor, images were acquired using the Maestro *In-Vivo* Imaging System (Cri Inc.). The following filter set was used: a band-path filter from 503 to 555 nm for excitation light and a long-pass filter over 645 nm for emission light. The tunable emission filter was automatically stepped in 10 nm increments from 600 to 800 nm at constant exposure times. The spectral fluorescence images consisting of spectra from autofluorescence and RFP were then unmixed, based on their known spectral patterns using commercial software (Maestro software; CRi).

### Image analysis

All images were analyzed using Image J software (http://rsb.info.nih.gov/ij/). First, using unprocessed images regions of interest (ROIs) were drawn within the tumor nodules depicted by the RFP images (true positive cancer foci) and in the normal adjacent small bowel, and then the average fluorescence intensity of each ROI was calculated. Next, we generated a fluorescence intensity curve using a time series of images. Subtracted images were created by subtracting the pre images (initial images before spraying gGlu-HMRG) from each of the post images (images after spraying gGlu-HMRG). Then, we calculated three parameters from each fluorescence intensity curve using subtracted images: maximum fluorescence signal (MF), wash-in rate (WIR), and area under the curve (AUC). MF is the maximum fluorescence signal observed during the entire dynamic images. WIR (fluorescence intensity/30 sec) is the maximum slope approaching the MF. AUC is the area measured under the time-fluorescence curve (Figure 1). Kinetic maps based on these three parameters, were created. For comparison of their utility for differentiating between cancer foci and background tissue at early time point, we calculated the tumor:small intestine ratio at 5 min after spraying gGlu-HMRG using following equation:

$$\text{T}:\text{SI}=\left({\text{TFI}}_{5\min}-{\text{SIFI}}_{5\min}\right)/\left|{\text{SIFI}}_{5\min}\right|$$

Where T:SI is the tumor:small intestine ratio, TFI is the fluorescence intensity of tumor at 5 min, SIFI is fluorescence intensity of small intestine at 5 min. We used the absolute value for the small intestine fluorescence intensity because baseline subtracted values may be negative in signal intensity.

### Statistical analysis

Statistical analysis was performed with JMP 10 software (SAS Institute, Cary, NC). We determined the differences in fluorescence intensity at 5, 10, 20, and 30 min after spraying gGlu-HMRG at various concentrations compared to the starting value and the changes in tumor:small intestine ratio compared to the unprocessed image using Dunnett's multiple comparison. The difference of fluorescence intensity on unprocessed images and three parameter images calculated from subtracted dynamic images between cancer foci and small intestine was determined at 5, 10, 20, and 30 min, respectively. For comparisons of fluorescence intensity between cancer foci and small intestine a two-sided Mann-Whitney's *U* test was employed. Differences of *p* < 0.05 were considered statistically significant.

## SUPPLEMENTARY MATERIALS FIGURES












