# Supplementary material for: Dynamic fluorescent imaging with the activatable probe, γ-glutamyl hydroxymethyl rhodamine green in the detection of peritoneal cancer metastases: Overcoming the problem of dilution when using a sprayable optical probe
Source: Oncotarget. 2016 Jun 7;7(32):51124–37. doi: 10.18632/oncotarget.9898 (PMC5239463; doi:10.18632/oncotarget.9898)
Supplement: Supplementary file 1 [file oncotarget-07-51124-s001.pdf]

Dynamic fluorescent imaging with the activatable probe, γ-glutamyl hydroxymethyl rhodamine green in the detection of peritoneal cancer metastases: Overcoming the problem of dilution when using a sprayable optical probe

Supplementary Materials

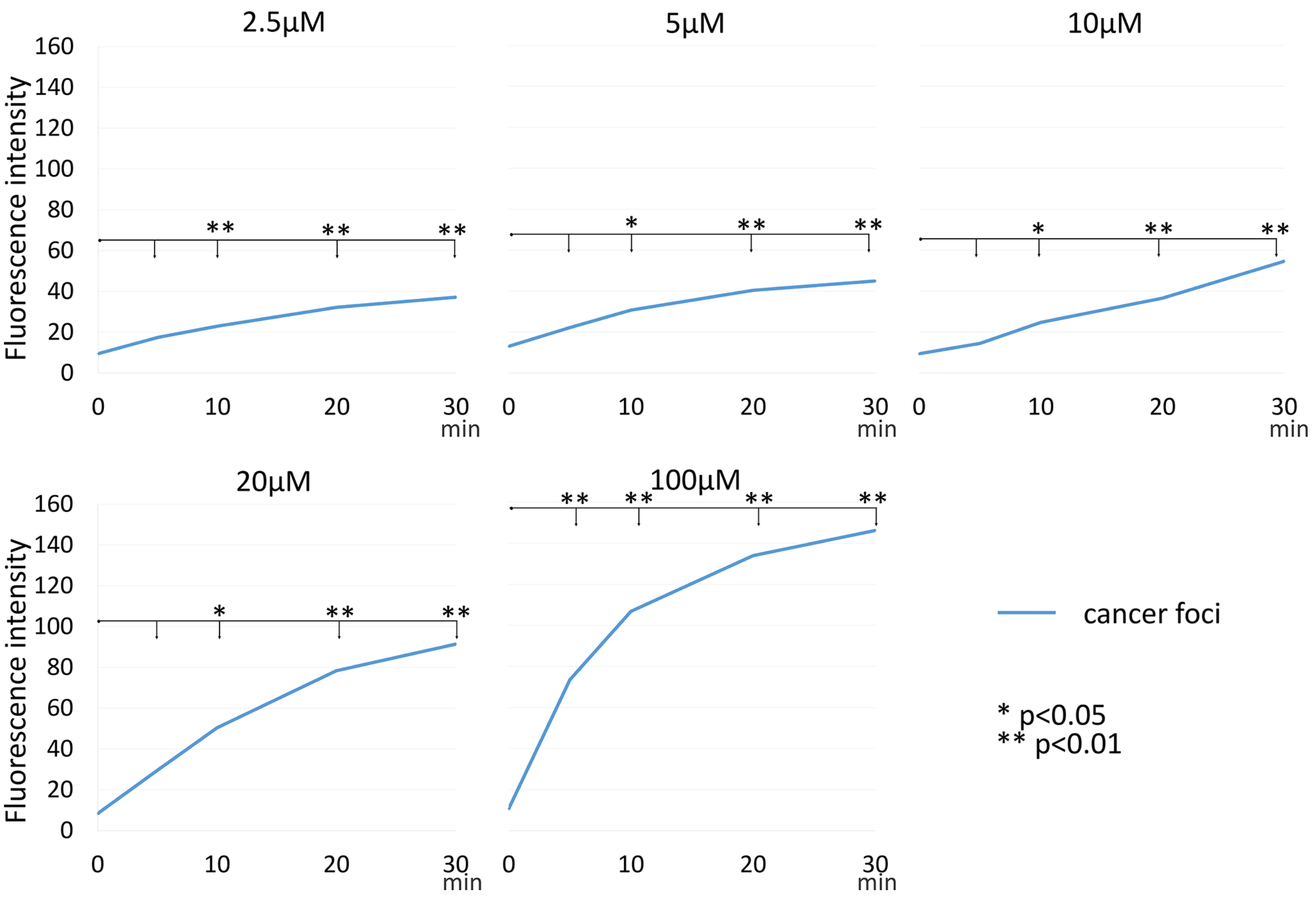

Supplementary Figure S1: Time fluorescence intensity curve of the cancer foci on unprocessed images. Difference at each time point compared to starting value was examined.

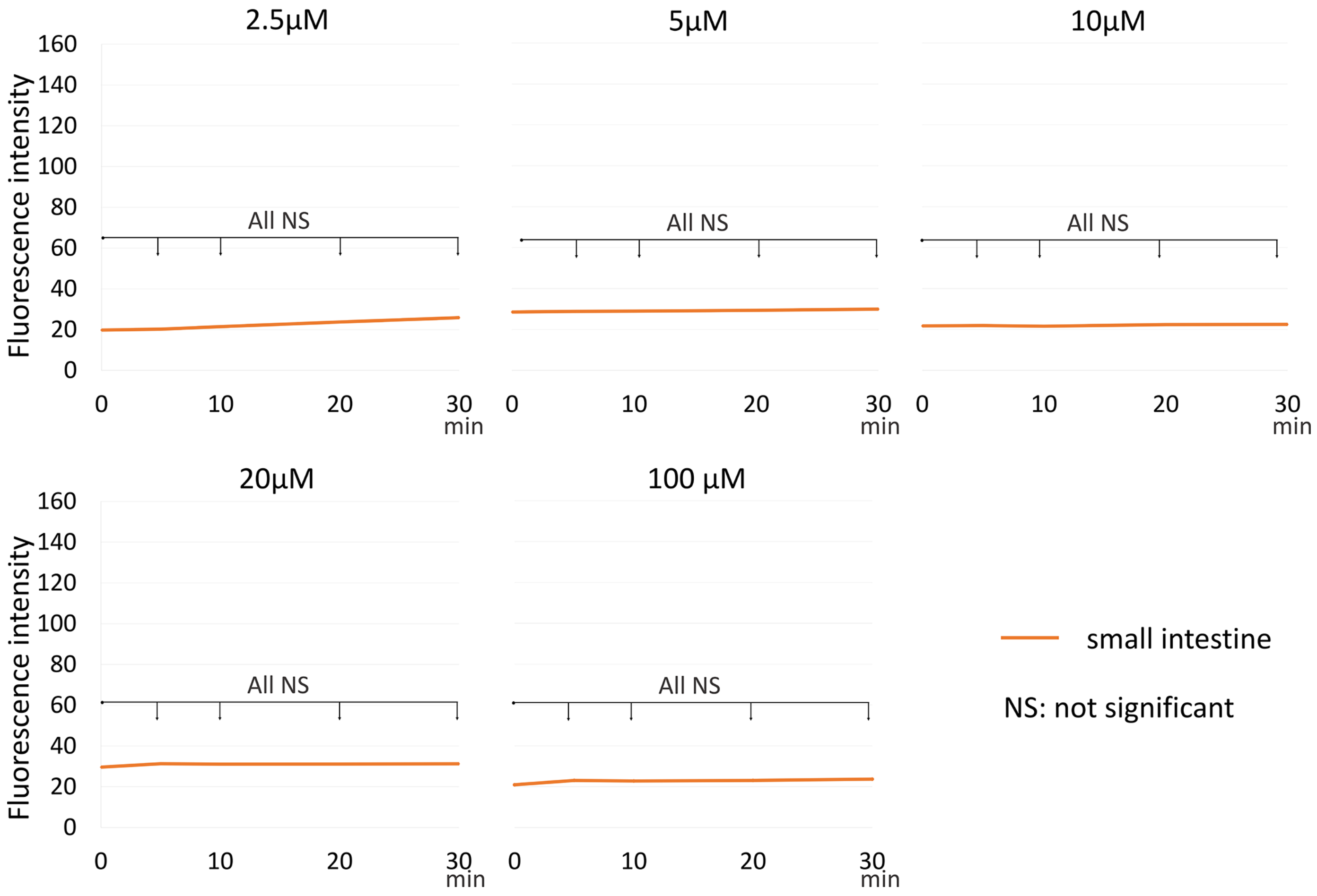

Supplementary Figure S2: Time fluorescence intensity curve of small intestine on unprocessed images. Difference at each time point compared to starting value was examined.

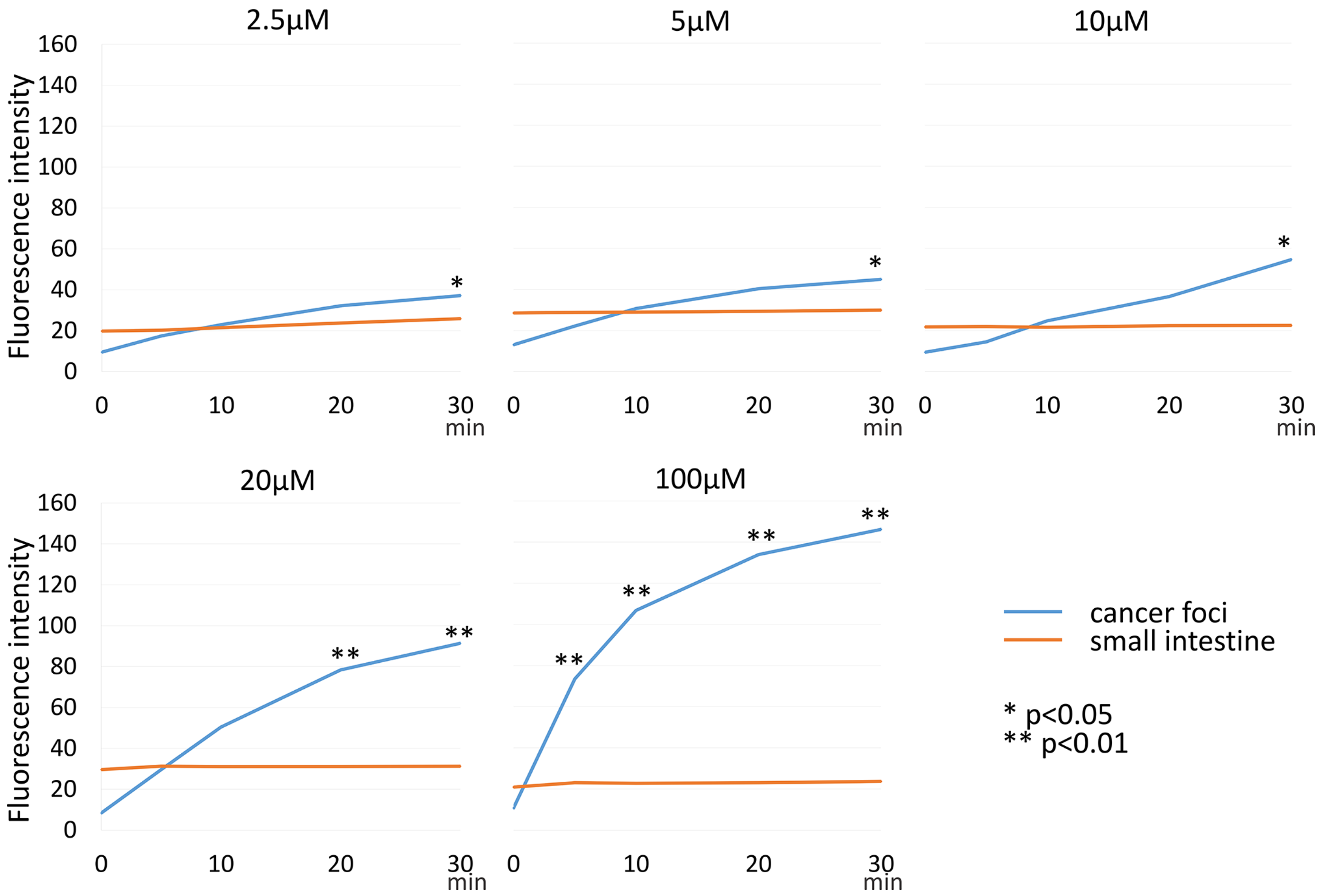

Supplementary Figure S3: Time fluorescence intensity curve of the cancer foci and small intestine on unprocessed image. Difference between cancer foci and small intestine was examined at each time point.

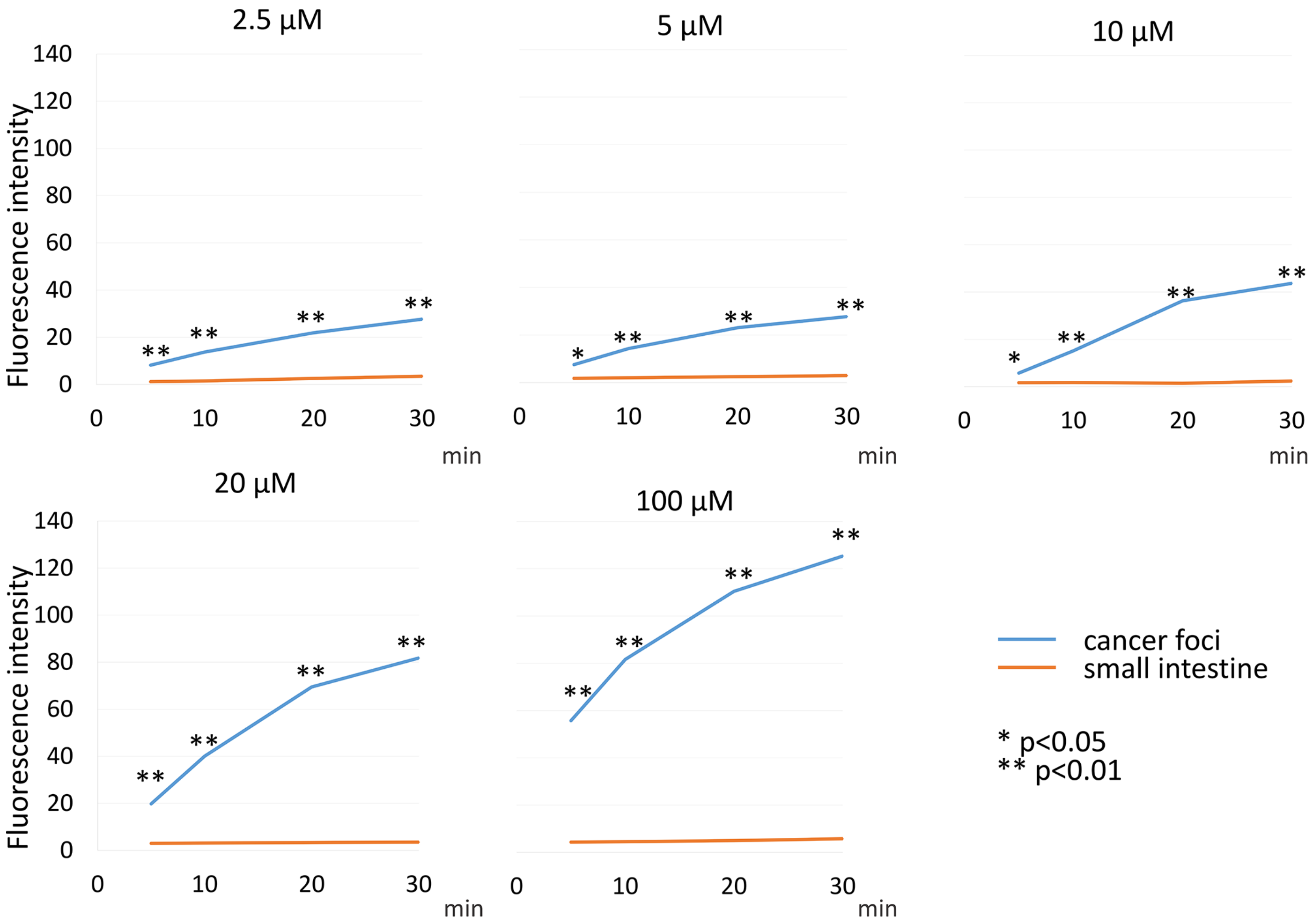

Supplementary Figure S4: Time fluorescence intensity curve of the cancer foci and small intestine on MF maps. Difference between cancer foci and small intestine was examined at each time point.

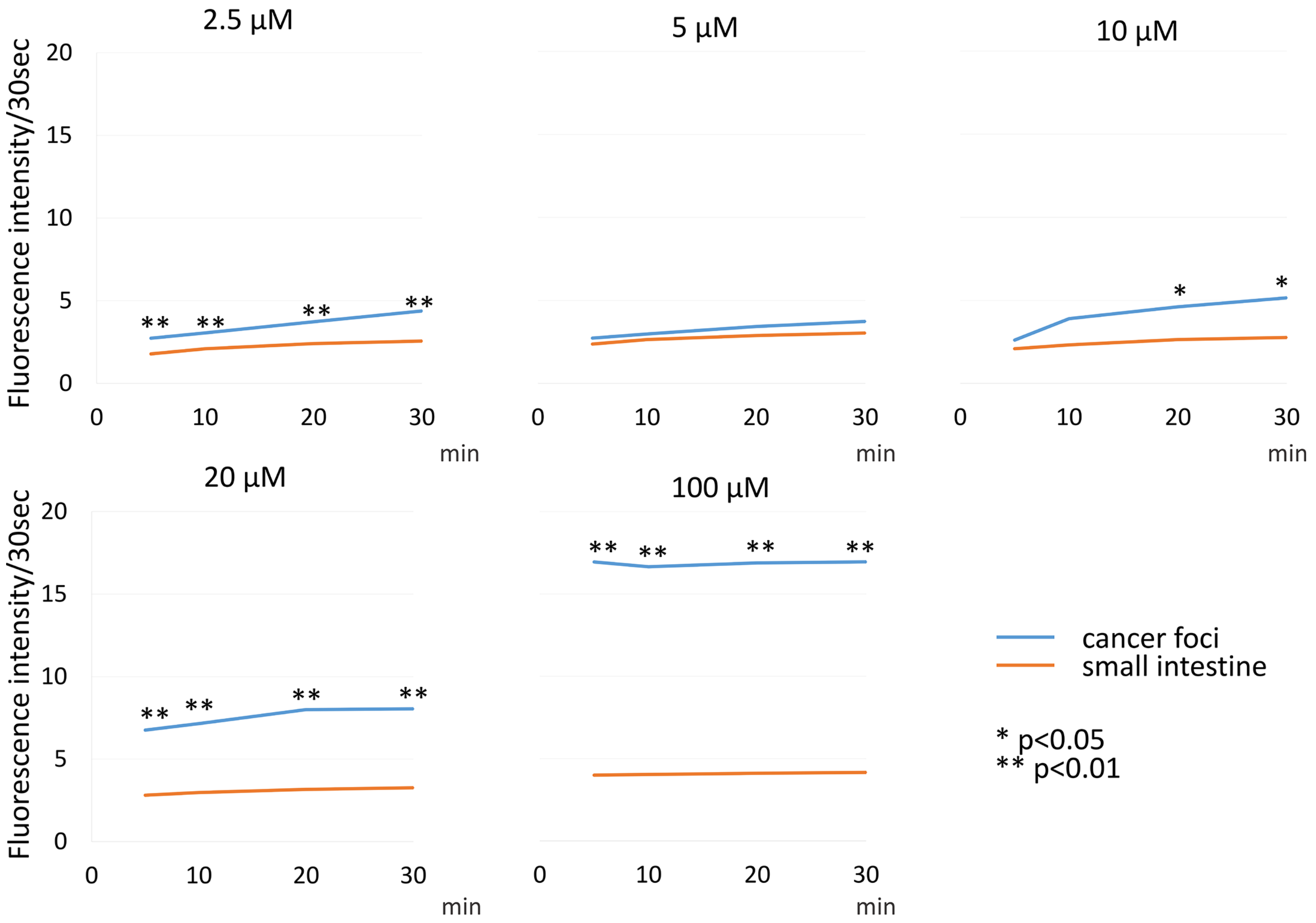

Supplementary Figure S5: Time fluorescence intensity curve of the cancer foci and small intestine on WIR maps. Difference between cancer foci and small intestine was examined at each time point.

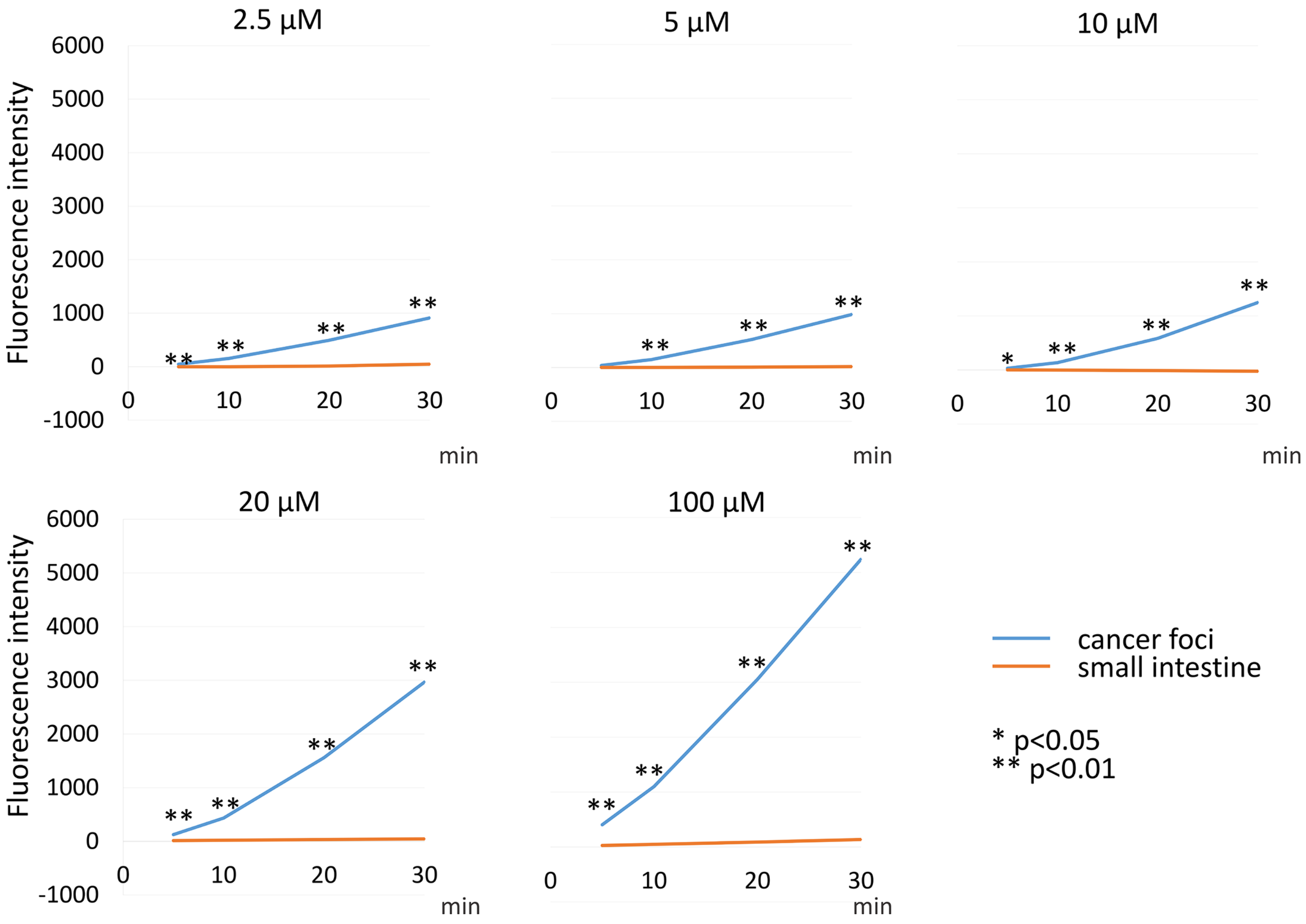

Supplementary Figure S6: Time fluorescence intensity curve of the cancer foci and small intestine on AUC maps. Difference between cancer foci and small intestine was examined at each time point.

Supplementary Video S1: Movie of serial unprocessed images after spraying 2.5 µM gGlu-HMRG. See Supplementary_Video_S1

Supplementary Video S2: Movie of serial unprocessed images after spraying 5 µM gGlu-HMRG. See Supplementary_Video_S2

Supplementary Video S3: Movie of serial unprocessed images after spraying 10 µM gGlu-HMRG. See Supplementary_Video_S3

Supplementary Video S4: Movie of serial unprocessed images after spraying 20 µM gGlu-HMRG. See Supplementary_Video_S4

Supplementary Video S5: Movie of serial unprocessed images after spraying 100 µM gGlu-HMRG. See Supplementary_Video_S5
